# Viability and Infectivity of *Plasmodium vivax* Gametocytes in Short-Term Culture

**DOI:** 10.3389/fcimb.2021.676276

**Published:** 2021-06-01

**Authors:** Glenda Quaresma Ramos, Djane Clarys Baia-da-Silva, Marcus Vinícius Guimarães Lacerda, Wuelton Marcelo Monteiro, Stefanie Costa Pinto Lopes

**Affiliations:** ^1^ Programa de Pós-Graduação em Medicina Tropical, Universidade do Estado do Amazonas, Manaus, Brazil; ^2^ Instituto de Pesquisa Clínica Carlos Borborema, Fundação de Medicina Tropical Dr. Heitor Vieira Dourado, Manaus, Brazil; ^3^ Instituto Leônidas & Maria Deane, FIOCRUZ, Manaus, Brazil

**Keywords:** malaria, membrane-feeding assay, *Plasmodium vivax*, transmission-blocking, gametocytes, culture

## Abstract

The control and elimination of malaria caused by *Plasmodium vivax* both represent a great challenge due to the biological aspects of the species. Gametocytes are the forms responsible for the transmission of the parasite to the vector and the search for new strategies for blocking transmission are essential in a scenario of control and elimination The challenges in this search in regard to *P. vivax* mainly stem from the lack of a long-term culture and the limitation of studies of gametocytes. This study evaluated the viability and infectivity of *P. vivax* gametocytes in short-term culture. The samples enriched in gametocytes using Percoll (i), using magnetic-activated cell sorting (MACS^®^) (ii), and using non-enriched samples (iii) were evaluated. After the procedures, gametocytes were cultured in IMDM medium for up to 48 h. Cultured *P. vivax* gametocytes were viable and infectious for up to 48 h, however differences in viability and infectivity were observed in the samples after 12 h of culture in relation to 0 h. Percoll-enriched samples were shown to be viable in culture for longer intervals than those purified using MACS^®^. Gametocyte viability after enrichment procedures and short-term culture may provide new avenues in the development of methods for evaluating *P. vivax* TB.

## Introduction

*Plasmodium vivax* is the leading malaria-causing species worldwide (World Health Organization, 2020) and some severe cases and mortality are reported in endemic are as in tropical and subtropical regions (Alexandre et al., 2010; Howes et al., 2016; Kotepui et al., 2020). *P. vivax* has unique biological characteristics, such as rapid gametocytogenesis that facilitates transmission and the development of latent forms, known as hypnozoites, which are responsible for relapses months after treatment (Bermúdez et al., 2018; Dhiman, 2019). These features present a great challenge in the control and elimination strategies of vivax malaria.

Gametocytes, which are the sexual stage of the parasite in humans and are the responsible for the transmission to the vector, have been the target of studies that could generate insights into the development of new strategies for blocking transmission (Gebru et al., 2017). However, current knowledge regarding gametocytes is the result of well-established *in vitro* studies of *Plasmodium falciparum* and experiments with *Plasmodium berghei* in rodents (Ngotho et al., 2019). In *P. vivax*, membrane-feeding assays (MFA) have been used to investigate the period of viability of gametocytes under different conditions (Vallejo et al., 2016; Pereira-Silva et al., 2021), the biological characteristics and dynamics (Vallejo et al., 2016) and candidates for transmission blocking (Pinilla et al., 2018; Fabbri et al., 2021).

Since *P. vivax* cannot be cultivated for long periods, *ex vivo* studies have provided several possibilities for analysis, however their focus is mainly on asexual stages, and few results on viability and infectivity are available regarding gametocytes. Thus, this study evaluates the viability and infectivity of gametocytes in a short-term *ex vivo P. vivax* culture.

## Materials and Methods

### Reagents

RPMI-1604, Human AB Serum, CF11 cellulose, Giemsa, xanthurenic acid and mercurochrome were obtained from Sigma-Aldrich (USA). Percoll was obtained from GE-Healthcare (USA). Iscove’s Modified Dulbecco’s Medium was obtained from Gibco (USA). The LS MACS columns and the MACS separator were obtained from Miltenyi Biotech (UK).

### Ethics Statement and Sample Collection

This study was approved by the Research Ethics Committee of FMT-HVD (CAAE approval 84151317.4.0000.0005). Adult volunteers (aged >18 years), presenting at *Fundação de Medicina Tropical Dr. Heitor Vieira Dourado* (FMT-HVD) with *P. vivax* malaria infection diagnosed by blood smears equal to or greater than two crosses (501–10,000 parasites/μl) (Ministério da Saúde, 2009) were invited to participate in the study. All study participants provided written informed consent. For each patient, approximately 9 ml of blood were collected *via* venipuncture and placed in a sterile heparinized Vacutainer^®^ tube.

### Blood Sample Processing

After recruitment, the sample was processed immediately. The blood was centrifuged at 400*g* for 5 min at 37 °C for plasma separation. Then, the pellet was resuspended with RPMI to a final 50% hematocrit and the leukocytes were removed using a cellulose column (Sriprawat et al., 2009). During all procedures, we used a hot plate inside the tissue culture hood and preheated solutions at 37 °C to prevent gametocyte exflagellation (Vera et al., 2015). For all analyses, the initial parasitemia and gametocytemia was determined by light microscopy (magnification 1,000×) in 50 fields (200 red blood cells per field) in Giemsa-stained thin blood film (Ministério da Saúde, 2009; Sriprawat et al., 2009).

### Percoll Gradient Purification

Gametocytes were enriched using Percoll 70% gradient according to Russell et al. (2011), with modifications. A 5 ml pellet was obtained after depletion of leukocytes and was adjusted to 50% hematocrit using RPMI, and 5-ml aliquots were carefully layered on a 5 ml 70% Isotonic Percoll and centrifuged at 1,200*g* for 15 min at 37°C, low acceleration and no brake. After the concentrated gametocytes formed on the Percoll interface were collected they were washed twice in 2 ml RPMI by centrifugation at 400*g*/5 min/37 °C. The percentage of parasites (parasites/total erythrocytes × 100) and gametocytes (gametocytes/parasites × 100) was determined on a Giemsa-stained thin film. Gametocytes were evaluated during 48 h (at 12, 24 and 48 h intervals) and 12 h (at 3, 6 and 12 h intervals) of cultivation.

### Magnetic Column Purification (MACS^®^)

Gametocytes were enriched using magnetic cell sorting (MACS) according to the methodology of Reuling et al. (2017), with modifications. After depletion of the leukocytes, 5 ml of blood was adjusted to a 50% hematocrit using RPMI. The MACS^®^-columns LS were coupled to the magnetic board and 3 ml of RPMI was run through the column once and, subsequently the blood sample. After running the sample, the column was washed with 3 ml of RPMI. This procedure was performed twice, until the eluent was free of red blood cells. Then, the column was detached and placed in a 15-ml centrifuge tube and 5 ml de RPMI was added, and the retained material was pressed through the column with the plunger. The sample was centrifuged (400*g*/5 min/37 °C) and the percentage of parasites (parasites/total erythrocytes × 100) and gametocytes (gametocytes/parasites × 100) was determined from the Giemsa-stained thin film. Gametocytes were analyzed for up to 24 h at intervals of 6, 12 and 24 h.

### Non-Enriched Samples

After leukocyte depletion, for each time period, 400 μl of pellet was cultured in 75 cm^2^ flasks and viability was evaluated for up to 48 h at intervals of 6, 12, 24 and 48 h.

### Gametocyte Culture

Enriched samples were diluted to a final parasitemia of 5% with non-infected O positive erythrocytes to yield gametocytemia, ranging from 0.1 to 0.5%, and cultured according to Rangel et al. (2018), with modifications. The samples were adjusted to 2% hematocrit using IMDM supplemented with 20% heat-inactivated human AB serum. For each time period, a total sample for MACS (21 µl ± 2 µl SEM) and Percoll (48 h: 38 µl ± 3 SEM; 12 h: 44 µl ± 5 SEM) were cultured in 5–15 wells (96-well plates–200 µl/well) under gas conditions of 5% CO_2_, 5% O_2_, 90% N_2_ and at a temperature of 37 °C.

### Gametocyte Viability

#### Viability Under Microscopy

Giemsa-stained thin films were prepared (2 µl) and the gametocyte viability was assessed by counting in a total of 50 fields (200 red blood cells per field). Gametocytes were classified as being viable or non-viable according to the morphology presented (**Supplementary Material Figure 1**). The slide was considered negative if no viable gametocytes were found in up 50–100 fields (Gebru et al., 2017).

#### Viability According to Exflagellation

Exflagellation tests were performed according to the methodology of Delves et al. (2013), with modifications. An aliquot of 400 ul of gametocyte culture was centrifuged at 400*g* for 5 min at 37 °C. The pellet cells were resuspended in 10 μl of RPMI medium supplemented with 10% heat-inactivated human AB serum and 100 μM of xanthurenic acid. After 10 min of incubation at room temperature (25°C), 10 μl were placed in a hemocytometer and the exflagellation centers were counted under a 10× lens at a reading of 25 fields.

#### Experimental Infection in *Anopheles aquasalis* Using a Membrane Feeding Assay (MFA)

Cultured gametocytes (4–14 wells for each time period) were centrifuged (400*g*/5 min/37°C), the medium was removed and the pellet obtained for samples enriched with MACS^®^ or Percoll was resuspended in 400 µl of uninfected fresh O+ blood to a final 40% hematocrit with heat-inactivated human AB serum. For the non-enriched samples, the gametocytes in the culture were centrifuged and the cell pellet was resuspended to a final 40% hematocrit with heat-inactivated human AB serum.

For each period analyzed, about 100 adult *An. aquasalis* females (3–5 years old) from the colony at Gerência de Entomologia/FMT-HVD were used. The *An. aquasalis* females were artificially fed through a glass feeder (diameter of 3 cm and a blood volume capacity of 1 ml) circuit coated with a Parafilm^®^ membrane and the blood was maintained at 37°C *via* a hose system connected to a thermal bath (Rios-Velásquez et al., 2013).

The sample was offered to the mosquitoes for a period of 45–90 min *via* MFA. Then, the fully engorged females were separated from the unfed females, transferred to rearing containers and maintained in the insectary at 26°C, 70–80% relative humidity and fed daily on a 10% sugar solution (Santana et al., 2019). After 7 days, the mosquitoes were dissected and the midguts were stained with 2% commercial mercurochrome and examined for the presence of oocysts in order to determine the infection rate (percentage of mosquitoes infected) and infection intensity (number of oocysts/mosquito) (Rios-Velásquez et al., 2013).

### Statistical Analysis

The statistical analyses were conducted using GraphPad Prism^®^ software, version 5.1. The normality of the data was assessed using the Kolmogorov–Smirnov test. Each time interval was compared with the reference value at 0 h. The statistical treatment of the gametocytes data and number of exflagellation centers were analyzed using ANOVA. For the infection rate and infection intensity, comparisons between groups were analyzed using the Mann–Whitney U test and the Kruskal–Wallis test, respectively. Differences were considered statistically significant when p <0.05.

## Results

### Viability and Infectivity by Percoll

**Figure 1:**
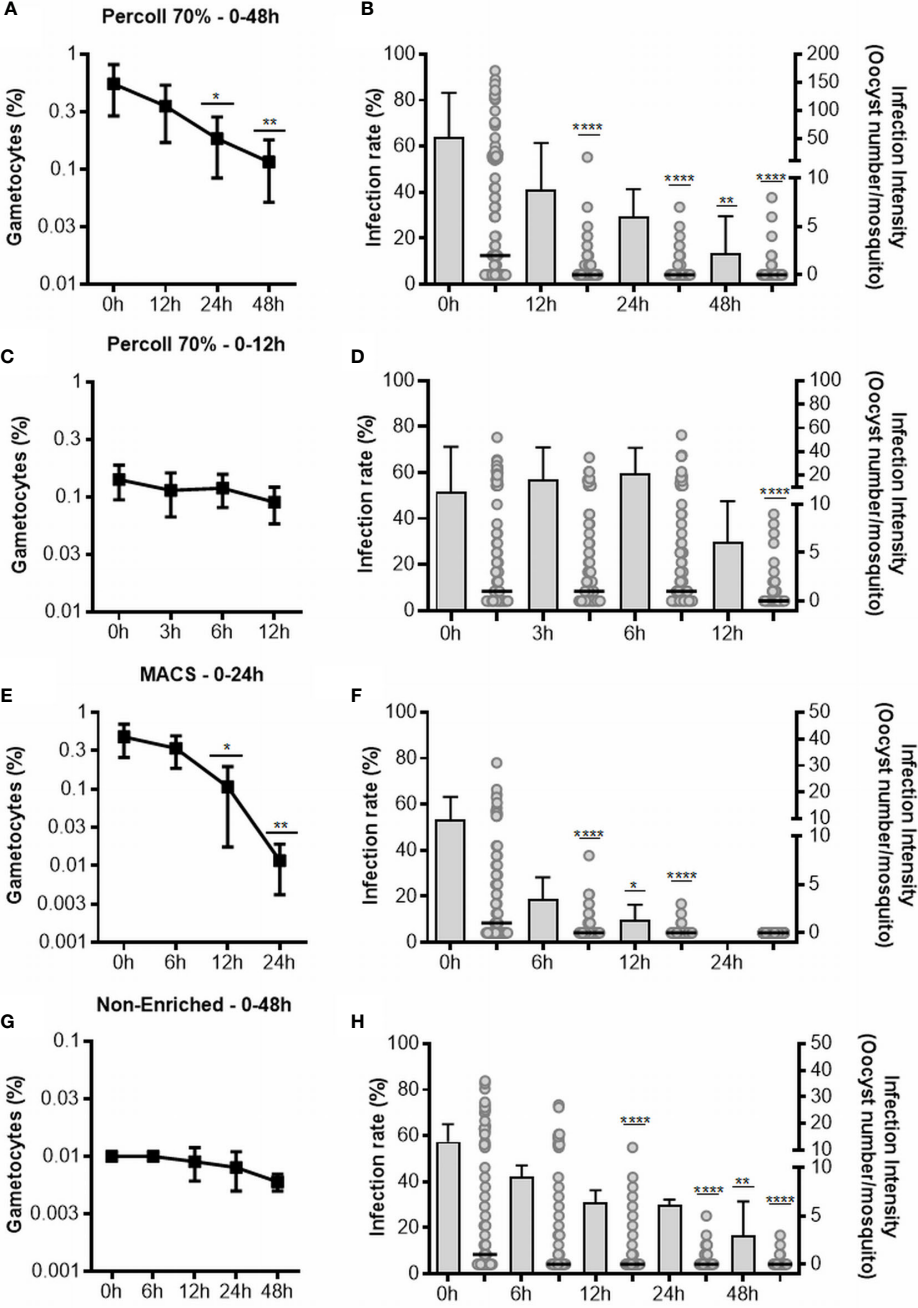
Gametocyte viability and infectivity in cultures enriched by Percoll after 48 **(A, B)** and 12 h **(C, D)**, enriched by MACS **(E, F)** and non-enriched **(G, H)**. The line graph in **(A, C, E, G)** represents the gametocytes in thin smears of culture stained with Giemsa. Each point represents the average of viable gametocytes counted for each interval, and vertical lines represent the standard error. Graphs **(B, D, F, H)** represent the infectivity. The infection rate is presented in bars as the mean and standard deviation, and the intensity of the infection is represented as the number of oocysts per single midgut (dots) with medians (horizontal black lines). Results shown for Percoll 48 h (n = 6), Percoll 12 h (n = 6), MACS (n = 6) and non-enriched (n = 5) are from independent experiments. Asterisks (*), (**), (****) represent significant differences (p <0.05, p <0.01 and p <0.0001, respectively) in relation to 0 h.

For enrichment using Percoll, we performed two sets of experiments. First, six isolates with initial gametocytemia of 0.09% ( ± 0.6% SEM) were processed. After Percoll, a mean gametocytemia of 15.25% ( ± 7% SEM) was obtained. Gametocyte cultures were analyzed for up to 48 h, at 12, 24 and 48 h intervals. The gametocyte counts decreased at the intervals of 24 and 48 h, when compared to 0 h (**Figure 1A**). Infectious gametocytes were present at all incubation periods. In relation to 0 h, significant decrease was found only at 48 h (p <0.0001) in infection rate and, in the intensity of infection, the number of oocysts decreased at all intervals (p <0.0001) (**Figure 1B** and **Table 1**).

**Table 1:** Experimental infection in *An. Aquasalis* by membrane feeding assay (MFA).

	Time		Number of mosquitoes examined (Total mosquitoes fed)	Infection Rate % (IQR)	Infection Intensity	
					Mean ± SEM	Median (Min–Max)
Percoll 70%	48 h					
		0 h	126 (241)	64.1 (51.4–87.2)	15.6 ± 3.2	2 (0–171)
		12 h	161 (249)	41.2 (25.1–57.1)	0.8 ± 0.1	0 (0–16)^c^
		24 h	111 (199)	29.3 (17.9–39)	0.5 ± 0.1	0 (0–7)^c^
		48 h	133 (241)	13.7 (0–31.4)^b^	0.3 ± 0.1	0 (0–8)^c^
	12 h					
		0 h	163 (272)	51.7 (40–65.2)	4.1 ± 0.6	1 (0–52)
		3h	125 (227)	57.1 (43.2–72.3)	3.1 ± 0.5	1 (0–35)
		6 h	185 (317)	60 (49–70)	3.7 ± 0.5	1 (0–54)
		12 h	149 (260)	30 (18.3–41.6)	0.7 ± 0.1	0 (0–9)^c^
MACS	24 h					
		0 h	168 (292)	53.4 (48–61.6)	3.4 ± 0.4	1 (0–31)
		6 h	135 (154)	18.7 (10.6–28.7)	0.4 ± 0.1	0 (0–8)^c^
		12 h	140 (239)	9.8 (4–14.5)^a^	0.1 ± 0.04	0 (0–3)^c^
		24 h	148 (229)	0	0 ± 0	0 (0–0)
Non-enriched	48 h					
		0 h	110 (183)	57.4 (51.4–64)	5.4 ± 0.8	1 (0–36)
		6 h	117 (189)	42.2 (37.2–47)	3.3 ± 0.5	0 (0–27)
		12 h	100 (175)	31.1 (27.5–35.4)	1.2 ± 0.2	0 (0–11)^c^
		24 h	118 (178)	29.9 (27.6–31.6)	0.5 ± 0.08	0 (0–5)^c^
		48 h	90 (161)	16.9 (3.1–31.5)^b^	0.1 ± 0.05	0 (0–3)^c^

IQR, interquartile range (25th and 75th percentile). ^a^p-value ≤0.05. ^b^p-value ≤0.01. ^c^p-value ≤0.0001.

In the 48-hour assay, we observed a significant decrease in the infectivity in all incubation periods evaluated. Therefore, we conducted another set of experiments with six isolates (initial gametocytemia of 0.02% ± 0.004% SEM), with the objective of evaluating gametocyte viability in periods shorter than 12 h. Thus, 8.2% ( ± 3.6% SD) of gametocytes were obtained by Percoll 70%. The gametocytes were maintained in culture for up to 12 h, with analysis at intervals of 3, 6 and 12 h. There was no difference in gametocyte counts at all the evaluated intervals in relation to 0 h (**Figure 1C**). When compared to 0 h, there was no change in the infection rate during 6 h of cultivation, but a decrease was found at 12 h, although it was not significant. For the intensity of infection, a significant decrease was observed only in the 12 h (p <0.0001) of incubation (**Figure 1D**, **Table 1**).

### Viability and Infectivity by MACS

Although Percoll had a high parasitemia enrichment profile (>40%), a large number of asexual forms were observed in the enriched material (**Supplementary Material Figure 2**). Thus, to obtain purer gametocyte material, we performed sample enrichment using MACS^®^. For this, six isolates with initial gametocytemia of 0.03% ( ± 0.01% SEM) were processed and we observed a mean gametocytemia of 59% ( ± 13.3% SEM) after purification. Considering the intervals analyzed in the Percoll experiments and final yield of the material obtained using MACS^®^, the cultures were analyzed for up to 24 h, at the intervals of 6, 12 and 24 h.

A reduction in the gametocyte counts was observed for the intervals of 12 h (p <0.05) and 24 h (p <0.01) when compared to 0 h (**Figure 1E**). Although morphologically viable gametocytes were observed by microscopy in all intervals, no oocysts were observed in mosquitoes fed with gametocytes cultured for 24 h (**Table 1** and **Figure 1F**). For the infection rates, we observed a reduction at intervals from 12 h (p <0.05) of incubation. Regarding infection intensity, at all analyzed intervals, a reduction in the number of oocysts was observed when compared with the control (p <0.0001), as shown in **Figure 1F** and **Table 1**.

### Viability and Infectivity in Samples Non-Enriched

To evaluate the viability of gametocytes in the culture without the enrichment procedures, five isolates with gametocytemia of 0.01% ( ± 0.002% SD) were cultured and analyzed up to 48 h, with analyses conducted at intervals of 6, 12, 24 and 48 h.

Analysis of the culture without any enrichment process indicated low gametocyte counts at all analyzed intervals; however, no effect on the gametocyte counts was found in any evaluated interval when compared to 0 h (**Figure 1G**). The infection rate was reduced when mosquitoes were fed with non-enriched samples cultured up to 48 h (p <0.01), while the intensity of infection was significantly diminished in the intervals of 12 (p <0.0001), 24 (p <0.0001) and 48 h (p <0.0001) when compared to 0 h (**Figure 1H** and **Table 1**).

### Gametocytes Viability by Exflagellation Assay

**Figure 2:**
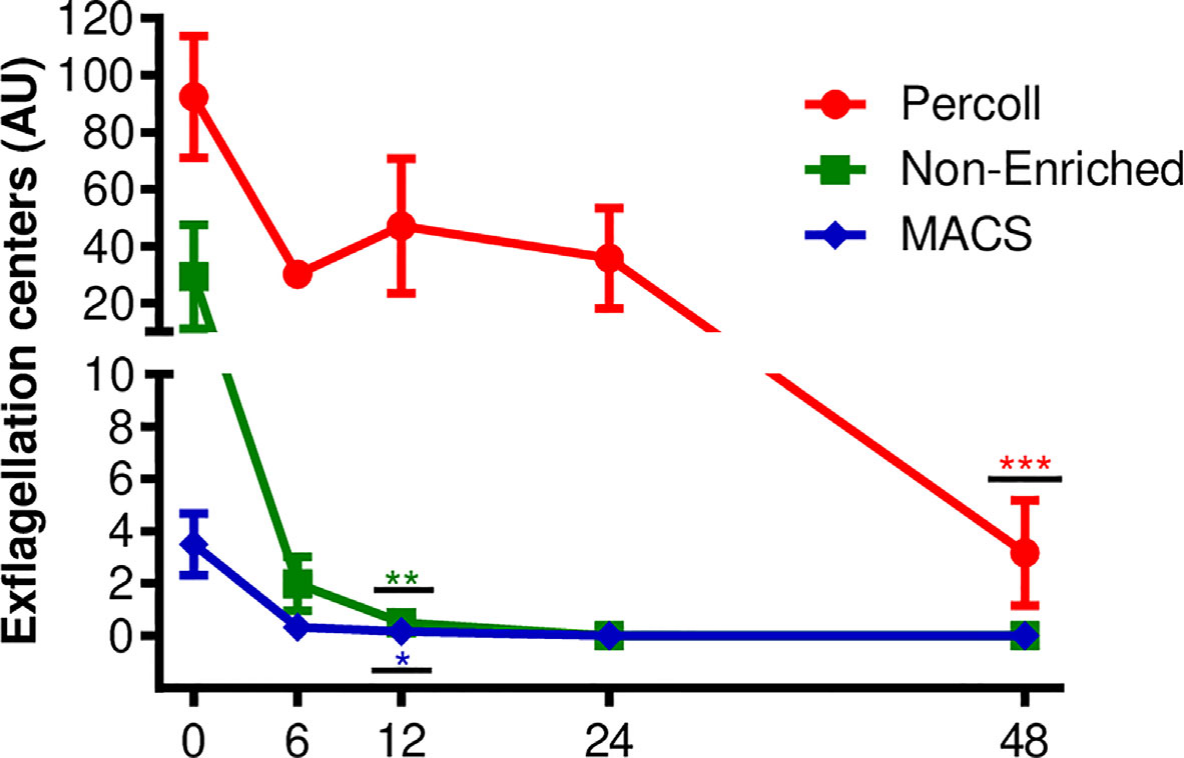
Viability by exflagellation assay. Number of exflagellation centers formed in *P. vivax* culture after Percoll 70% or MACS enriched processes and non-enriched samples. Each point represents the average number of exflagellation centers counted at each time interval, vertical lines represent the standard error. Data are from six independent experiments. Asterisks *p <0.05, **p <0.01, ***p <0.001 represent significant differences (using one-way ANOVA) compared to the control (0 h).

For the exflagellation assay, six isolates with initial gametocytemia of 0.03% ( ± 0.01% SEM) were evaluated. After purification using Percoll or MACS, the gametocytemia was enriched and means of 14.83% ( ± 5% SD) and 20% ( ± 3.4% SD), respectively, were obtained under culture conditions, the exflagellation was analyzed for up 48 h, at 6, 12, 24 and 48 h intervals. As shown in **Figure 2**, samples purified using the Percoll 70% method showed centers of exflagellation at all the intervals evaluated, with fluctuation of exflagellation centers over time. Statistical differences were found only at 48 h (p <0.001). Exflagellation in the samples processed using MACS^®^ and the samples without enrichment showed exflagellation in up to 12 h of cultivation (p <0.05 and p <0.01, respectively) (**Figure 2**).

## Discussion

The viability of the gametocytes is essential for the success of parasite transmission, and it is also essential when designing transmission blocking studies (Gebru et al., 2017). In this study, *via* an *ex vivo* culture, we tested the viability period in cultures of gametocytes using different methods for enrichment of parasites and non-enriched samples.

Enrichment methods have been used previously in order to obtain samples with a high concentration of parasites that allow standardization for experimental analysis (Rangel et al., 2018), since, in addition to heterogeneity, *P. vivax* infections present low parasitemia (Howes et al., 2016). Furthermore, in the majority of infections, gametocytes may be present in low densities (Bousema & Drakeley, 2011). Vera et al. (2015), in an assay using *P. vivax*, showed that Percoll and MACS^®^ were suitable for purification of gametocytes, with no significant difference between the two in terms of purification efficacy.

Percoll 70% has been used to obtain ring stages (Russell et al., 2012; Saliba & Jacobs-Lorena, 2012) and early gametocytes in *P. falciparum* (Saliba & Jacobs-Lorena, 2012) and, enrichment using MACS provided material with stages >30 h (Ribaut et al., 2008). In our analysis, we observed that, after purification using Percoll 70% and MACS, the gametocytes were infectious, nevertheless the period of viability in culture showed differences. In samples enriched using Percoll and samples without enrichment, the gametocytes were able to infect mosquitoes for up to 48 h; however the infection rate and intensity decreased significantly after 12 h. In MACS^®^ groups, the viability was observed up to 12 h, with significant decreases after 6 h of culture.

In the literature, the life span and infectivity of gametocytes in non-falciparum species can vary from 6 to 12 h after maturation (Hawking et al., 1971; Gautret & Motard, 1999; Armistead et al., 2018). Transcriptome studies in *P. vivax ex vivo* cultures showed that ring conversion to immature gametocytes started after 20 h of culture, with maturation ranging from 17–20 h (Obaldia et al., 2018). In this study, the differences in the interval of viability between methods may be related to a possible maturation of gametocytes presented in non-enriched and Percoll-enriched samples at intervals after 12 h of cultivation. However, in our study, as well as infectivity, we evaluated the viability of gametocytes using microscopy of blood smears and, unlike *P. falciparum*, the elucidation of gametocytes at different stages of maturation is still not morphologically well known (Baro et al., 2017).

Despite being restricted to the evaluation of male gametocytes, exflagellation assays are extensively used to evaluate the functionality of male gametocytes in cultures (Ruecker et al., 2014; Leba et al., 2015). In our exflagellation assays, Percoll 70% presented the highest number of exflagellation events, which were also observed at all the time intervals.

The non-enriched samples showed exflagellation centers for up to 12 h, despite their infectivity at 48 h. The results maybe a limitation of the assay and are related to the low parasitemia that the non-enriched samples present and, therefore, the absence of exflagellation centers in the evaluated microscopy fields. Unlike the exflagellation assay, the MFA experiments with non-enriched samples showed similarity with the Percoll 70% results in infectivity for up to 48 h. These results maybe provide assays with less complex techniques and processing time, as seen in enrichment methods (Russell et al., 2012).

In our experiments, there were some limitations: i. a low number of mosquitoes analyzed can be observed, and this is mainly due to the percentage of fully engorged ( ∼ 40%) which can be affected by several factors related to the membrane assay, such as temperature, starvation period, feeding time and mosquito density per cage (Bousema et al., 2012; Vallejo et al., 2016a). However, the mean specimens dissected per assay ( ∼ n = 24) is in consonance with other studies using the same anopheline specimen from the colony and presents means of infectivity (compared to time 0h) similar (Rios-Velásquez et al., 2013; Vera et al., 2015; Baia-Da-Silva et al., 2018; Martins-Campos et al., 2018; Pinilla et al., 2018); ii. due to the logistics, the MFAs were not carried out in paired assays and the exflagellation assay was not compared with infected mosquitoes, which makes it difficult to fully compare the methods.

Overall, our study showed that, in *ex vivo* cultures, gametocytes were viable and infectious for up to 48 h, with significant differences from 12 h. Recently, our group has evaluated methods for testing transmission blocking compound candidates (Fabbri et al., 2021) and, despite being well used, a direct membrane feeding assay (DMFA) can present limitations such as the lack of an exposure time capable of evaluating possible gametocidal activity. In our analysis, we observed that gametocytes cultured within 6 h can be used initially to screen for new agents with anti-gametocyte action and, as such, this allows direct validation of infectious gametocytes. In a malaria control and elimination scenario, the study of gametocyte viability and infectivity are important in order to explore new blocking strategies, and these results contribute by providing new perspectives in the development of the necessary tools for the investigation of agents for blocking transmission.

## Data Availability Statement

The raw data supporting the conclusions of this article will be made available by the authors, without undue reservation.

## Ethics Statement

The studies involving human participants were reviewed and approved by Research Ethics Committee of Fundação de Medicina Tropical Dr. Heitor Vieira Dourado (CAAE approval 84151317.4.0000.0005). The participants provided their written informed consent to participate in this study.

## Author Contributions

GR performed and acquired all the experimental data, and drafted the manuscript. DB, ML, WM and SL were responsible for reviewing and editing the final manuscript. ML, WM and SL supported funding acquisition. SL supervised the work. All authors contributed to the article and approved the submitted version.

## Funding

This study was funded by Conselho Nacional de Desenvolvimento Científico e Tecnológico (CNPq, grant 442849/2019-2), Fundação Oswaldo Cruz (Programa INOVA Novos Talentos), Coordenação de Aperfeiçoamento de Pessoal de Nível Superior (CAPES; Finance Code 001). WM acknowledges funding support from Fundação de Amparo à Pesquisa do Estado do Amazonas (PAPAC 005/2019, Universal Amazonas/006/2019, PRÓ-ESTADO and Posgrad calls). ML and WM are CNPq fellows.

## Conflict of Interest

The authors declare that the research was conducted in the absence of any commercial or financial relationships that could be construed as a potential conflict of interest.
