# Supplementary material for: Viability and Infectivity of Plasmodium vivax Gametocytes in Short-Term Culture
Source: Front Cell Infect Microbiol. 2021 Jun 1;11:676276. doi: 10.3389/fcimb.2021.676276 (PMC8204544; doi:10.3389/fcimb.2021.676276)
Supplement: Supplementary file 1 [file DataSheet_1.pdf]

**Supplementary Material**

**Viability and infectivity of *P. vivax* gametocytes in short-term culture**

Glenda Quaresma Ramos, Djane Clarys Baia-da-Silva; Marcus Vinícius Guimarães Lacerda, Wuelton Marcelo Monteiro, Stefanie Costa Pinto Lopes*

* Correspondence: Stefanie Costa Pinto Lopes: stefaniecplopes@gmail.com

**Supplementary Figures**

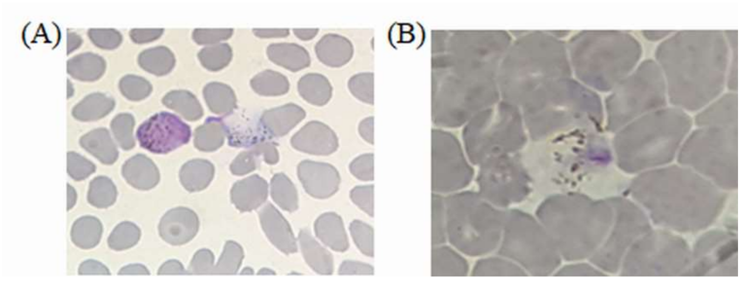


**Supplementary Figure 1.** Viability observed under microscopy. A) Viable gametocyte and B) Non-viable gametocytes

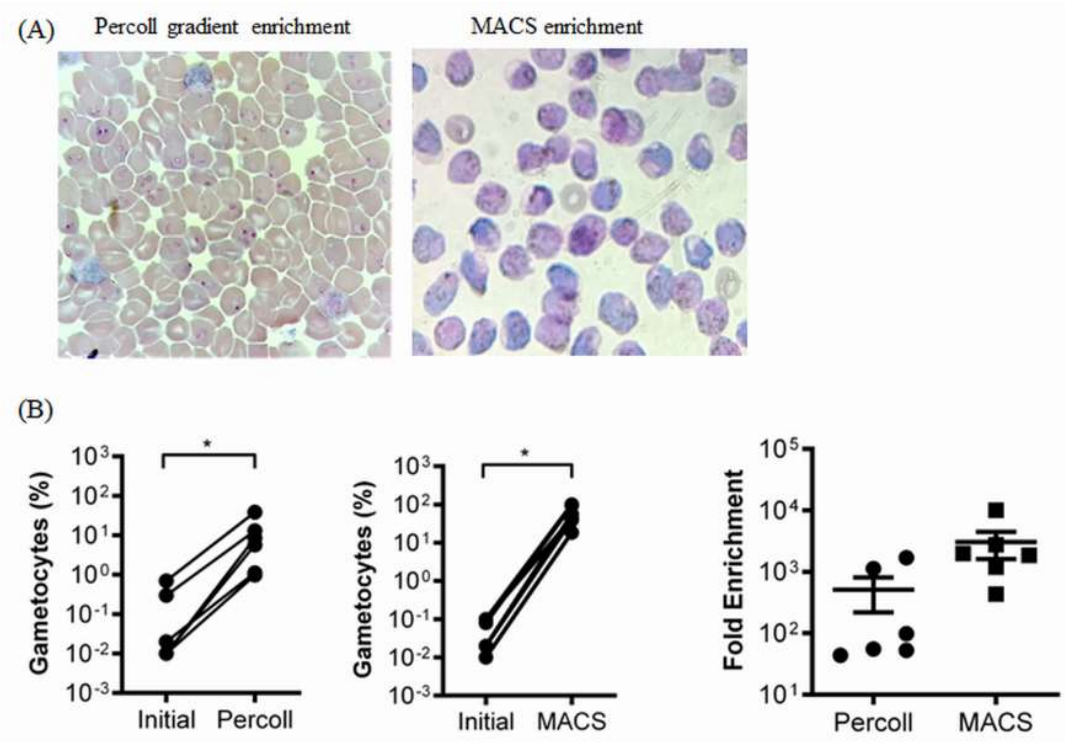


**Supplementary Figure 2.** Enrichment methods: (A) Giemsa staining of purified material from Percoll or MACS samples. (B) Initial and enriched gametocytes (Paired Student's t test statistics; **p*< 0.05) and Fold gametocytes enrichment of isolates using the different methods. Each dot represents the isolates (n=6) and bars represents mean (larger) and standard error.
